# Xenosurveillance reflects traditional sampling techniques for the identification of human pathogens: A comparative study in West Africa

**DOI:** 10.1371/journal.pntd.0006348

**Published:** 2018-03-21

**Authors:** Joseph R. Fauver, James Weger-Lucarelli, Lawrence S. Fakoli, Kpehe Bolay, Fatorma K. Bolay, Joseph W. Diclaro, Doug E. Brackney, Brian D. Foy, Mark D. Stenglein, Gregory D. Ebel

**Affiliations:** 1 Department of Microbiology, Immunology, and Pathology, Colorado State University, Fort Collins, Colorado, United States of America; 2 Liberian Institute for Biomedical Research, Charlesville, Liberia; 3 United States Naval Medical Research Unit No. 3, Cairo, Egypt; Fundaçao Oswaldo Cruz, BRAZIL

## Abstract

**Background:**

Novel surveillance strategies are needed to detect the rapid and continuous emergence of infectious disease agents. Ideally, new sampling strategies should be simple to implement, technologically uncomplicated, and applicable to areas where emergence events are known to occur. To this end, xenosurveillance is a technique that makes use of blood collected by hematophagous arthropods to monitor and identify vertebrate pathogens. Mosquitoes are largely ubiquitous animals that often exist in sizable populations. As well, many domestic or peridomestic species of mosquitoes will preferentially take blood-meals from humans, making them a unique and largely untapped reservoir to collect human blood.

**Methodology/Principal findings:**

We sought to take advantage of this phenomenon by systematically collecting blood-fed mosquitoes during a field trail in Northern Liberia to determine whether pathogen sequences from blood engorged mosquitoes accurately mirror those obtained directly from humans. Specifically, blood was collected from humans via finger-stick and by aspirating bloodfed mosquitoes from the inside of houses. Shotgun metagenomic sequencing of RNA and DNA derived from these specimens was performed to detect pathogen sequences. Samples obtained from xenosurveillance and from finger-stick blood collection produced a similar number and quality of reads aligning to two human viruses, GB virus C and hepatitis B virus.

**Conclusions/Significance:**

This study represents the first systematic comparison between xenosurveillance and more traditional sampling methodologies, while also demonstrating the viability of xenosurveillance as a tool to sample human blood for circulating pathogens.

## Author summary

Infectious diseases continue to be a burden on mankind, particularly in the developing countries of the tropics. Recognition of pathogen transmission in humans is a crucial step to thwarting epidemics of these pathogens. However, sampling human blood or tissue is invasive and logistically difficult. Xenosurveillance takes advantage of the blood-feeding behavior of mosquitoes to sample human blood for the presence of infectious disease agents. In this study, we aimed to compare xenosurveillance to a more traditional sampling method to assess the usefulness of this technique in field settings where it could potentially be beneficial. DNA and RNA next generation sequencing followed by an in-house bioinformatic pipeline identified viruses and parasites of human origin in blood collected by either mosquitoes or finger-stick. Xenosurveillance produces samples of comparable quality to finger-stick blood collections while alleviating many of the difficulties of direct human sampling. This study suggests xenosurveillance can be a complimentary strategy for infectious disease surveillance in low-resource areas.

## Introduction

Emerging and reemerging infectious diseases (EID) pose a major public health threat throughout the world [1]. The burden of infectious disease, from persistent infections [2] to intermittent outbreaks [3], is especially high in developing countries of the tropics [4]. The amount of disability-adjusted life years (DALYs) and years of life lost (YLL) due to infectious diseases has decreased globally in the last decade, however in areas of the tropics, particularly in sub-Saharan Africa (SSA), infectious diseases still account for the majority of DALYs and YLL [5]. Despite this, the burden of infectious disease in SSA is likely underestimated due to misdiagnoses stemming from inadequate healthcare infrastructure with limited availability of diagnostic tests, procedures, and surveillance [6, 7]. These factors contribute to an environment conducive for EIDs to go unrecognized until they have caused substantial morbidity and mortality in a human population. This is highlighted by the recent outbreak of Ebola virus in West Africa [8, 9], and demonstrates the need for an improved diagnostic and surveillance framework in SSA.

Almost two-thirds of all infectious diseases of humans are of zoonotic origin [10]. Of these, 60% are EIDs [10, 11]. Pathogen emergence has been described as a step-wise process consisting of three parts; pre-emergence, localized emergence, and pandemic emergence [12]. Pre-emergence describes pathogen transmission in natural reservoir populations where some disturbance results in pathogen expansion within natural populations, an increase in pathogen host range (non-human), and/or pathogen spread to a new geographical area. Localized emergence is characterized as pathogen spillover into human populations with restricted animal-human and/or human-human transmission. Finally, pandemic emergence refers to global spread of the pathogen through human-human transmission, or sustained transmission through the appropriate vector [12].

Due to the threat of pandemic emergence, global surveillance programs that are aimed, in some capacity, at detecting zoonotic pathogens have increased in the last decade [13–18]. Active sampling of wildlife and domestic animals has identified pathogens that may be capable of causing pandemics (i.e. pre-emergence) [19–24]. However, substantial physiological, ecological, and evolutionary barriers exist to pathogen host switching, and the majority of animal pathogens cannot become zoonotic [12, 25–28]. In order to recognize pathogens prior to pandemic emergence, it would be helpful to sample pathogens that are circulating in human populations at the stage of localized emergence. Active sampling of human blood and/or tissue is the ideal strategy to detect localized emergence, however, human sample acquisition can be invasive, costly, logistically challenging, and requires institutional review board (IRB) approval. Consequently, developing non-invasive and cost effective strategies to collect human samples for pathogen screening are necessary.

Mosquitoes are efficient samplers of human blood. We have previously described xenosurveillance, a surveillance technique that makes use of the hematophagous behavior of some arthropods to survey vertebrates for the presence of pathogens [29, 30]. These studies demonstrated that blood meals from *Anopheles gambiae* mosquitoes are sufficient samples from which to detect viruses, bacteria, and parasites using quantitative PCR (qPCR) and reverse transcription PCR (qRT-PCR) along with next generation sequencing (NGS) in laboratory and field experiments.

It remains to be determined whether xenosurveillance is directly comparable to traditional sampling techniques (e.g. finger-sticks or venous blood draws) under field conditions. Accordingly, we compared xenosurveillance with blood collected via finger-stick in two villages in northern Liberia to determine whether these methods detect the same pathogens. Pathogens were detected by NGS in pooled xenosurveillance samples and finger-stick blood samples and then confirmed with qPCR and qRT-PCR to make prevalence estimates in the two sample types. Moreover, composition and count of NGS reads aligning the detected pathogens were compared between the two methods. Our results confirm that xenosurveillance and finger-stick methods for surveillance detect the same pathogens from a field setting in rural West Africa.

## Methods

### Ethics statement

Human subject sampling was approved by the Institutional Review Board at Colorado State University (CSU) (protocol 15-5896H) and by the National Research Ethics Board of Liberia (NREB-0017-15) in partnership with the Liberian Institute for Biomedical Research (LIBR). A local public health worker explained the details of the study and acquired signatures or thumb prints from individuals providing consent. The IRB protocol allowed for thumb prints to be used as informed consent for illiterate individuals. Informed consent was first obtained from the heads-of-households, followed by individual members of the household. Parents and guardians provided consent on behalf of children within the household. Body temperature was collected from each consenting individual within the household at the beginning of the study. All febrile patients (based on a body temperature ≥ 38°C) were offered a SD Bioline Malaria Antigen rapid diagnostic test (Standard Diagnostics, Republic of Korea) to determine the presence of malaria parasites [31]. Patients with a positive test were offered treatment with artemisinin-based combination therapy by a nurse and public health worker per WHO Standards [32]. No adverse events were reported.

### Study location, sampling, mosquito processing and storage

**Fig 1:**
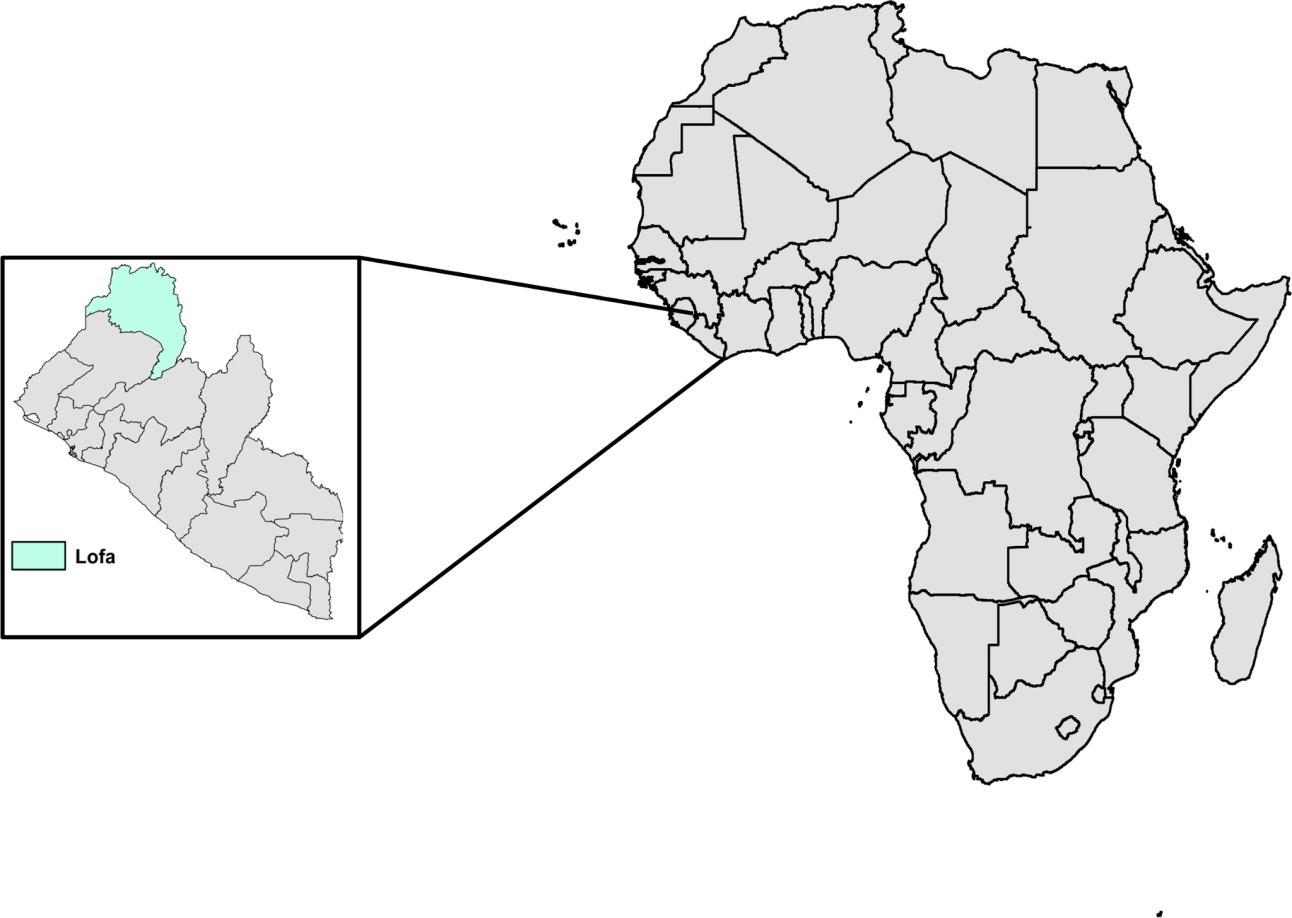
Households from two villages in northern Liberia were enrolled into the study. The two study villages were located in rural Lofa County, Liberia and made up of ~30 households each. Both Village A and Village B were visited by the research team consisting of researchers from CSU and LIBR, as well as a nurse and local public health worker. A total of 23 households from Village A and 20 households from Village B were enrolled into the study. Map made with free online tool at https://mapchart.net/.

Prior to the study, researchers from CSU and LIBR traveled to northern Liberia in order to recruit villages into the current study. Multiple villages in Lofa County, Liberia were visited (Fig 1). Individual households within two villages were enrolled. In an attempt to make our sample size as large as possible, each house in each village was visited, although not every house chose to participate. Upon enrollment, all members of the household provided blood via finger-stick performed by a local nurse recruited into the study. The finger surface was swabbed with an ethanol wipe prior to blood collection. Finger prick blood (hereafter referred to as human dried bloodspots (H-DBS)) was pipetted onto CloneSaver FTA cards (GE Healthcare, USA), and immediately soaked in 25μL of RNA*later* (ThermoFisher Scientific, USA) in order to facilitate diffusion of blood into the FTA card, as well as stabilize the nucleic acid. Body temperature was collected upon enrollment and during each sampling period, however enrollment was not contingent upon presenting as febrile.

Following enrollment of households, villages were visited every other day for up to two weeks to collect engorged female mosquitoes as previously described with slight modification [29]. Mosquitoes were aspirated from inside of houses with InsectaZookas (Bioquip, USA) prior to sunrise in order to collect mosquitoes that fed the previous night. Aspiration collections were sorted by date and location. Collections were transported to the LIBR research station in Bolahun, Liberia, where mosquitoes were identified using morphological keys [33]. Abdomens were dissected from blood fed mosquitoes using forceps and blood meals were applied to FTA cards as previously described [30], with the addition of soaking blood in RNA*later*, hereafter referred to as mosquito-dried bloodspots (M-DBS). FTA cards containing both H/M-DBS were placed in multi-barrier pouches (GE Healthcare, USA) containing desiccant beads to reduce humidity and prevent microbial growth. Samples were kept at 4°C until stored on icepacks and shipped to CSU. Pouches containing H/M-DBS were stored at -80°C until further processing.

### Library preparation for next generation sequencing

Laboratory processing of samples has been described previously [30]. Next Generation Sequencing (NGS) was used to assess H/M-DBS samples for the presence of pathogen-derived nucleic acid. RNA NGS was performed on H/M-DBS samples from a single household in Village A. The remaining samples from Village A were subject to DNA NGS. RNA NGS samples were separated into two pools by location and sample type. Total nucleic acid extraction on H/M-DBS was performed using the Mag-Bind Viral DNA/RNA kit (Omega Bio-tek, USA) and eluted into 50μL of water. Due to low RNA yield from H/M-DBS, samples were pooled by volume for both RNA and DNA NGS. The H-DBS pool was composed of 25μL of total RNA from each H-DBS, whereas the M-DBS pool was composed of 10μL of total RNA from each M-DBS. Each pool was DNAse treated using DNA-*free* DNA Removal Kit (Invitrogen, USA). Pools were purified using a 2x ratio of RNA clean XP beads (Beckman Coulter, USA) and eluted into 30μL of water. In order to increase reads of potential pathogen nucleic acid, an in-house protocol that uses gene specific primers and RNAse H to deplete pools of rRNA was employed. Following rRNA depletion, double-stranded DNA (cDNA) was created from the remaining RNA. First-strand synthesis was performed using the SuperScript III First-Strand Synthesis System following manufactures protocol (Invitrogen, USA). 2^nd^ strand cDNA synthesis was conducted immediately following 1^st^ strand synthesis using a Klenow Fragment (3”-5”exo-) (New England Biolabs, USA). For DNA NGS, 10μL of total DNA for H and M-DBS were pooled by sample type.

Library preparation inputs were quantified fluorometrically using the Qubit 3.0 High Sensitivity DNA assay (ThermoFisher, USA). cDNA created from RNA NGS pools was not quantifiable using a Quibit due to low concentration. Both RNA and DNA NGS samples were subject to library preparation with Nextera XT following manufactures protocol with slight adjustment (Illumina, USA). Due to low concentrations from RNA NGS samples, the Amplicon Tagment Mix was diluted 10-fold in order to tagment cDNA [34]. A dual indexing strategy was used for DNA NGS pools, and single end indexing was use for RNA NGS pools. Unique Illumina indices were incorporated to each pool using Kapa Library Amplification Kit for Illumina (Kapa BioSystems, USA). Individual libraries were quantified using the NEBNext Library Quant Kit for Illumina (New England Biolabs, USA). If necessary, libraries were re-amplified using the Kapa Library Amplification Kit for Illumina in order to achieve the necessary quantity for sequencing. Libraries were diluted to equal concentrations and pooled by volume for denaturing and loading. DNA NGS samples were sequenced on an Illumina MiSeq platform using a 600 cycle (2x300 reads) MiSeq v3 reagent kit at the CSU NGS facility. RNA NGS samples were sequenced on an Illumina NextSeq platform using a 150 cycle (1x150 reads) NextSeq Mid-Output Kit at the CSU NGS facility.

### Sequencing analysis

The goal of the sequencing analysis was to search for reads and contigs aligning to human derived pathogens. The processing pipeline is similar to that described in [35] (found online at https://github.com/stenglein-lab/taxonomy_pipeline). High level taxonomic assessment of individual reads was initially performed using BLASTn- megablast tool against the nt database with evalue = 1e-8 [36] to determine the composition of sequencing pools. An in-house script (tally_hits.pl, available at https://github.com/stenglein-lab/stenglein_lab_scripts) was used to count the number of reads aligning to eukaryotes, bacteria, and viruses (S1 File). Further, this process was also used to determine the number or reads aligning to the human and *An*. *gambiae* genomes.

Following the taxonomic assessment pipeline, contiguous sequences (contigs) that produced quality alignments of interest were viewed in Geneious 10.2.2 [37]. Multiple contigs aligning to pathogens were discovered. These contigs were used as to guide for further investigation. This initial dataset was produced following the filtering of reads aligning to mosquito genomic and rRNA. Human genomic DNA was removed using Bowtie2 version 2.2.5 with parameters–sensitive-score-min C,60,0 [38] for further analysis. Reference files for GBV-C (Accession #KM670099.1), HBV (Accession # KU736927.1) and *Plasmodium falciparum* (Genebank# 256198) were downloaded from NCBI GenBank. Whole genome FASTA files for multiple species of nematode worms, including *Brugia malayi* (BioProject #PRJNA10729, *Dracunculus medinensis* (BioProject #PRJEB500), *Enterobius vermicularis* (BioProject #PRJEB503), *Onchocerca volvulus* (BioProject #PRJEB513), *Loa loa* (BioProject # PRJNA60051), *Wuchereria bancrofti* (BioProject #PRJNA275548), and *Caenorhabditis elegans* (BioProject # PRJNA13758) were downloaded from WormBase (http://parasite.wormbase.org/index.html) and concatenated into a single FASTA file. Reference FASTA files were indexed using the–build option in Bowtie2. Following removal of mosquito genomic DNA, mosquito rRNA, and human genomic DNA, paired (DNA Seq) or single (RNA Seq) end reads were aligned to indexed reference files using–x and–very-sensitive options in Bowtie2 and exported as .SAM files using the–S option. Aligned .SAM files were converted to .BAM files and sorted to their reference genes using the view and sort options in SAMtools [39]. Individual reads that aligned were then assessed visually and with the BLASTn -megablast tool under previously listed parameters. NGS reads have been deposited in the NCBI Short Read Archive with links to BioProject PRJNA432355. All sequencing samples have been de-identified.

### PCR confirmation

In order to validate data obtained through NGS, we designed species-specific PCR primers to 1) confirm the presence of our target of interest in individual DBS, and 2) determine the prevalence of the detected pathogens from our samples. Primers were designed using the Primer3 software version 2.3.4 in Geneious (S1 Table) [40]. The presence of GBV-C was determined from individual DBS using a one-step reverse transcription polymerase chain reaction (RT-PCR) kit (Qiagen, Germany). The sequencing reaction was run on a 1% agarose gel to visualize the amplified product. Samples that produced visible bands were sent for Sanger sequencing using the forward primer at Quintarabio labs (USA). Chromatogram files were then aligned to the reference genome in Geneious 10.2.2 to confirm specificity. The presence of HBV was determined from individual DBS using the iTaq Universal One-Step RT-qPCR Kit (Bio-Rad, USA) containing SYBR green on a real-time PCR platform. Positive samples were sequenced and confirmation analysis was performed as stated above.

### Phylogenetic analysis

Phylogenetic trees for both GBV-C and HBV were built using a neighbor-joining method with no out group in Geneious 10.2.2. Full length genomes were downloaded from NCBI using the nucleotide feature in Geneious 10.2.2 and aligned. NGS reads aligning to GBV-C and HBV were mapped to their appropriate alignment, and the longest contig from each data set, a 491 nt segment aligning to the NS5B region of GBV-C and a 542 nt segment spanning the C and P gene of HBV, were used for the analysis. Both contigs were made of reads derived from H and M-DBS, as the overlapping regions had > 99% nucleotide identity. These regions were extracted from the alignments and used as the input sequences to build phylogenetic trees.

## Results

### Enrollment information and sample collection

Two villages in Northern Liberia were enrolled in our study (Fig 1). Village A was sampled on 6 occasions, while Village B was sampled on 3 occasions. Upon enrollment, no individuals presented as febrile. Throughout the course of sampling, two individuals presented as febrile based on a body temperature ≥ 38°C. Both individuals were positive for *P*. *falciparum* infection based on the results of a SD Bioline Malaria Antigen rapid diagnostic test and were provided artemisinin-based combination therapy. *Anopheles gambiae* sensu lato was the most commonly collected species of mosquito from within households in both villages, making up over 80% of mosquitoes collected during the study (Table 1). Few other taxa of mosquitoes were collected from inside households. Of the other taxa, *Aedes* and *Culex* mosquitoes were the most common genera, with 9 and 3 females, respectively. The vast majority of *An*. *gambiae* mosquitoes collected contained a full blood meal, indicating they fed the previous night. Village A was slightly more populous than Village B, resulting in a higher number of people enrolled into the study. As well, a greater number of blood fed *An*. *gambiae* mosquitoes were collected from households in Village A (Table 1). Following successive collection of mosquitoes from Village B, substantially more H-DBS were collected than were M-DBS. Previous experience aspirating mosquitoes from inside houses in West Africa implied this was an unexpectedly low number. Consequently, H/M-DBS from Village A were used for the remainder of our study.

**Table 1:** Summary of enrollment and sampling data.

Sample	Village A	Village B
Households	23	20
H-DBS^a^	105	80
Mosquitoes Aspirated	198 (81%)^c^	55 (87%)
M-DBS^b^	161	48

^a^ Human Dried Bloodspot

^b^ Mosquito Dried Bloodspot

^C^ Number in parenthesis refers to percentage of aspirated mosquitoes that were *An*. *gambiae* and bloodfed

### Sequencing analysis

RNA sequencing was performed on a subset of samples on an Illumina NextSeq instrument. This subset was made up from samples collected from an individual household in Village A. This location was selected because it had a higher than average number of individuals enrolled (7) and it produced the highest number of M-DBS (34). Prior to quality control and host filtering, each DBS produced over 1.8 million reads on average (S2 Table). Following quality control and host filtering, an average of ~50,000 reads remained per H-DBS and ~11,000 per M-DBS. The greatest reduction in reads was seen after the dataset was collapsed to unique reads, removing PCR duplicates (S2 Table).

The remaining samples were subjected to DNA sequencing on an Illumina MiSeq instrument. In total, 98 H-DBS were pooled separately from the remaining 127 M-DBS. The H-DBS pool produced over 700,000 reads while the M-DBS pool produced almost 900,000 (S2 Table). The greatest reduction in reads from DNA sequencing samples was observed following the filtering out of the human and mosquito genomic reads (S2 Table). For both RNA and DNA sequencing, the volume of each pool sequenced was based on the number of DBS in any pool, with the goal of obtaining similar number of reads per DBS.

### Taxonomic assessment

**Fig 2:**
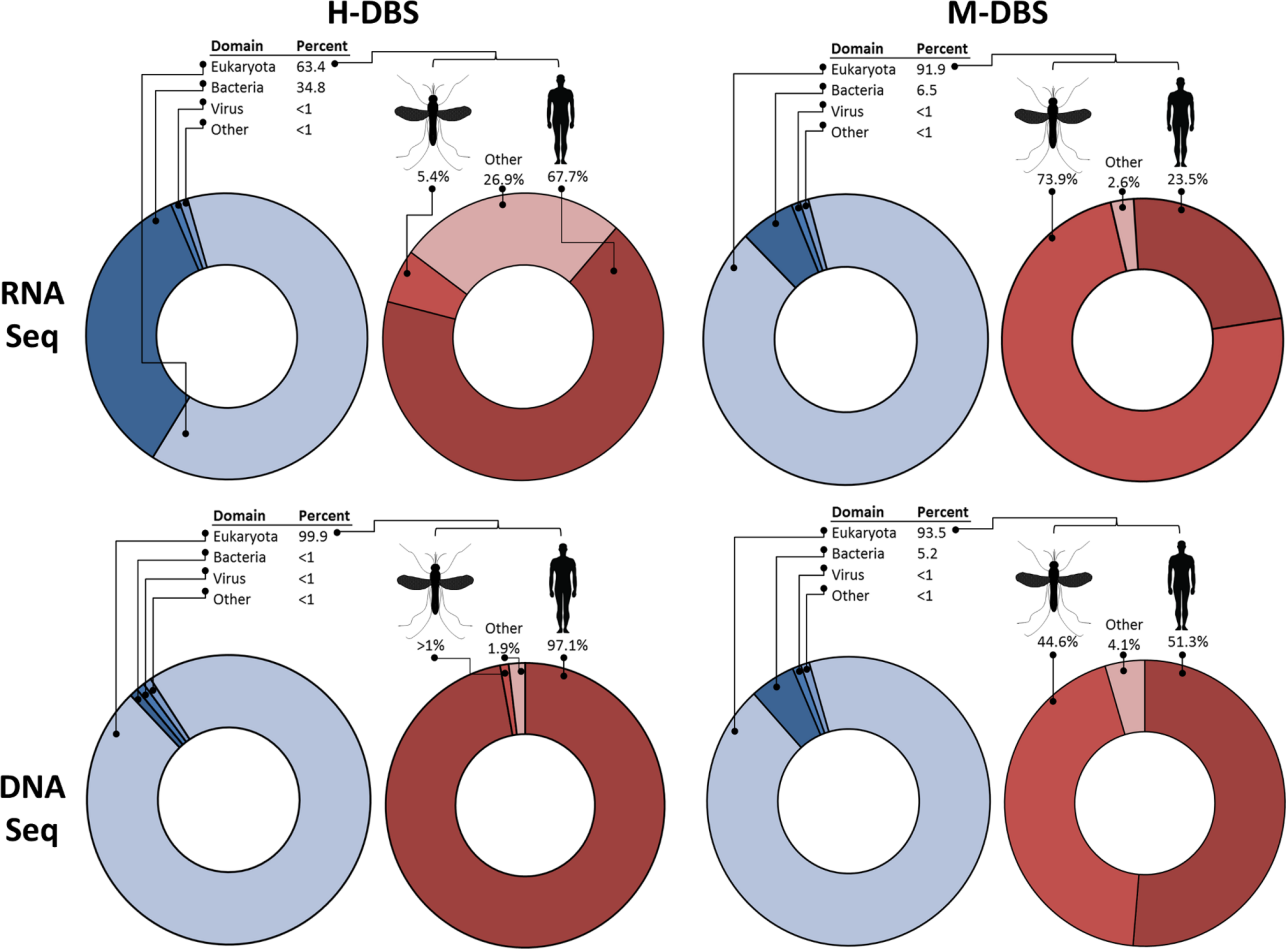
The majority of reads from M-DBS and H-DBS, for both RNA and DNA NGS pools, align to host nucleic acid. The taxonomic makeup of each sequencing pool was determined using the Blastn -Megablast tool with individual sequencing reads as input. Blue circles show taxonomic make up at the level of kingdom for all reads in each pool. Red circles show taxonomic makeup from a subset of reads aligning to eukaryotes. While the most reads in H-DBS aligned to human nucleic acid, reads from M-DBS aligned to both human and mosquito nucleic acid. Reads to human pathogens were detected in both H/M-DBS in the remaining reads.

The majority of reads from all of the sequencing pools aligned to eukaryotic organisms, and subsequently to host nucleic acid (Fig 2, S1 File). The most common reads from H-DBS were human derived, while the most common reads from M-DBS were derived from human and *An*. *gambiae*, indicating the likely origin of the blood meal. While host nucleic acid made up a preponderance of the sequencing libraries, reads of bacterial, viral, and parasitic origins were identified. These included two human viruses, GB-virus C (GBV-C, Family Flaviviridae) and Hepatitis B virus (HBV, Family Hepadnaviridae), *P*. *falciparum*, and multiple species of parasitic worms.

**Fig 3:**
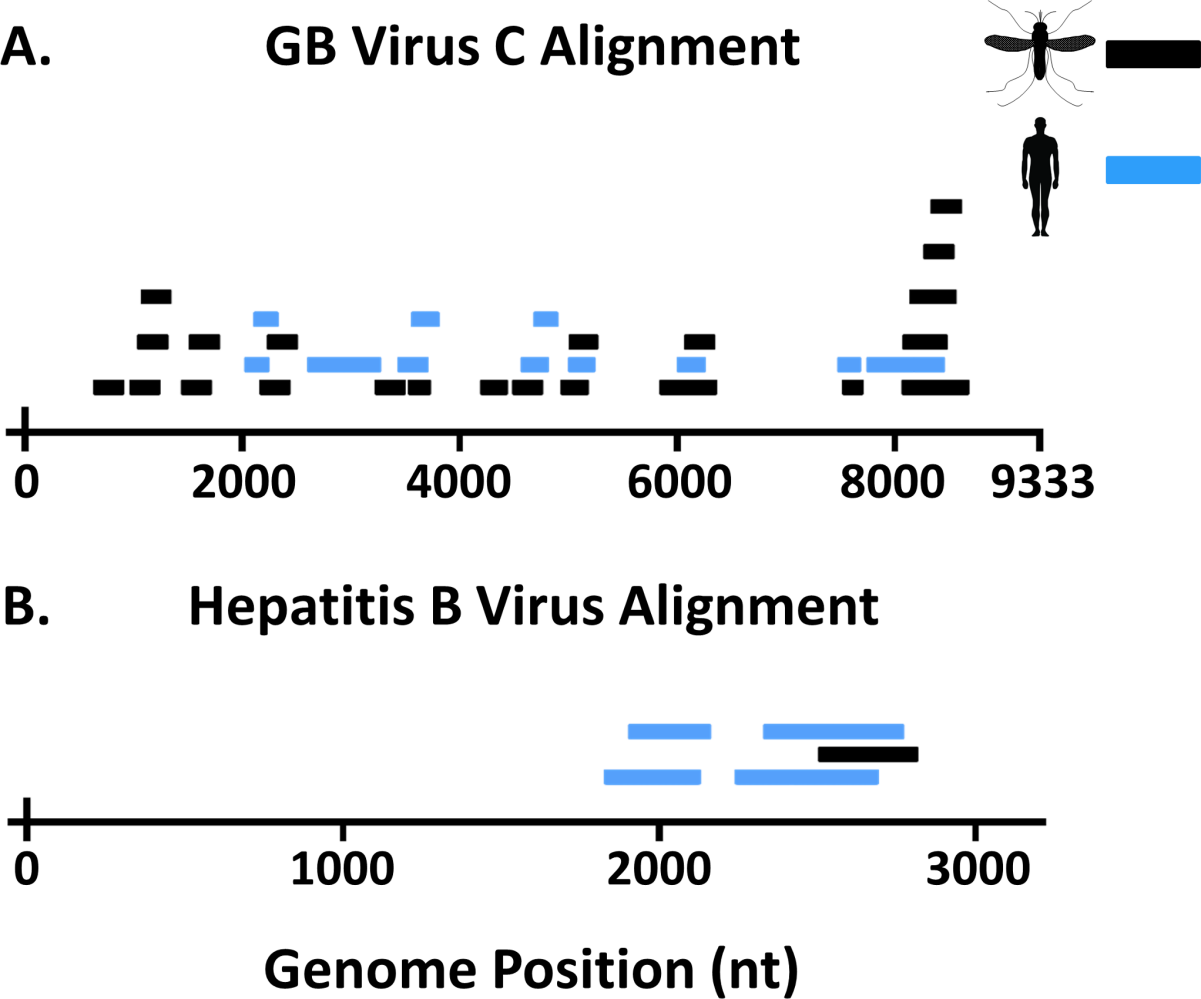
H and M-DBS showed reads aligning to human viruses at similar levels. A) RNA sequencing revealed multiple reads from pools of both H and M-DBS spanning the GB virus C genome. B) Hepatitis B virus reads were discovered from DNA sequencing pools of both H and M-DBS. For both viruses, reads from M-DBS were comparable in quantity and quality to reads from H-DBS.

**Fig 4:**
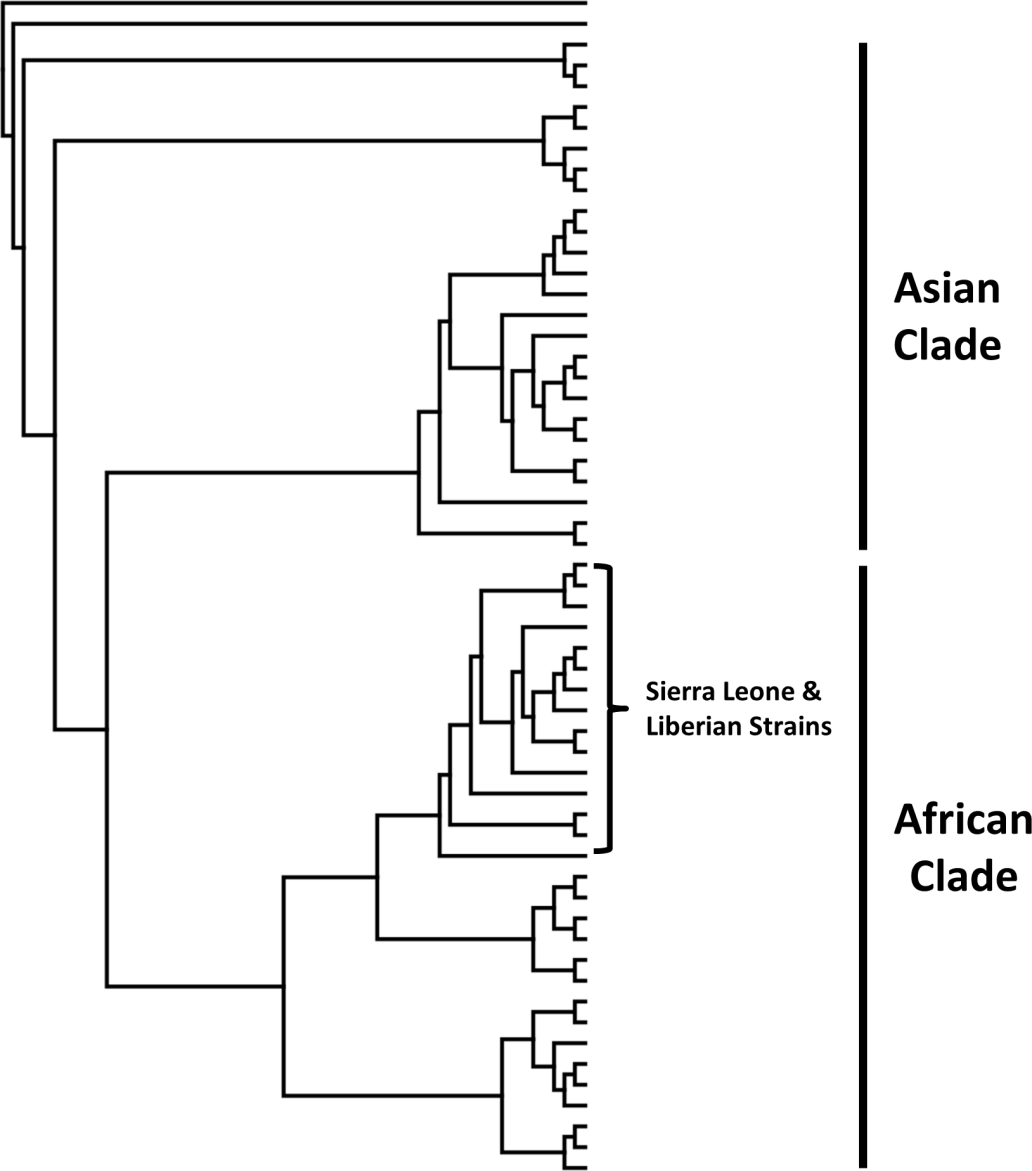
GBV-C sequences from H and M-DBS cluster phylogenetically with GBV-C strains from Sierra Leone and Liberia. The longest contig assembled to GBV-C, a 491 n.t. segment, was used as input to create a phylogenetic tree using a neighbor-joining method. The red line corresponds to input sequence generated from NGS data. See S1 Fig for accession numbers corresponding to each GBV-C strain used in the analysis.

Individual contigs aligning to a West African strain of GBV-C were produced from both H-DBS and M-DBS from our RNA sequencing datasets [41], indicating the virus was detected in human and mosquito samples collected from the same household. This genome was then used to make a reference index and individual reads from both H-DBS and M-DBS sequencing dataset were aligned (Fig 3A). In total, 15 and 28 individual reads aligned to GBV-C from H-DBS and M-DBS, respectively. On average, these reads aligned with over 90% pairwise nucleotide similarity. Mean individual read length was 128 nucleotides, and these reads spanned ~40% of the genome (Table 2). The overlapping reads/contigs from M-DBS and H-DBS shared 99% pairwise nt identity. A phylogenetic analysis using over 50 partial GBV-C sequences resulted in two major clades that grouped by geographic origin of virus sequences (Fig 4, S1 Fig). The sequences identified in this study grouped most closely with GBV-C sequences identified from humans in West Africa [41].

**Table 2:** NGS reads aligning to human viruses.

**RNA NGS**					
Sample	Nucleic Acid	# DBS	Reads aligning to GBV-C	% N.T. similarity GBV-C^a^	% Genome Coverage^b^
H-DBS Pool	RNA	7	15	91.8	18.8
M- DBS Pool	RNA	34	28	93.8	26.5
**DNA NGS**					
Sample	Nucleic Acid	# DBS	Reads aligning to HBV	% N.T. similarity HBV^c^	% Genome Coverage^d^
H-DBS Pool	DNA	98	6	98.9	24.4
M- DBS Pool	DNA	127	2	99.3	8.5

a West African strain of GB virus C (Accession #KM670099.1), N.T. (Nucleotide)

b 39.8% of total genome covered

c African strain of HBV (Accession #KU736927.1), N.T. (Nucleotide)

d 25.8% of total genome covered

**Fig 5:**
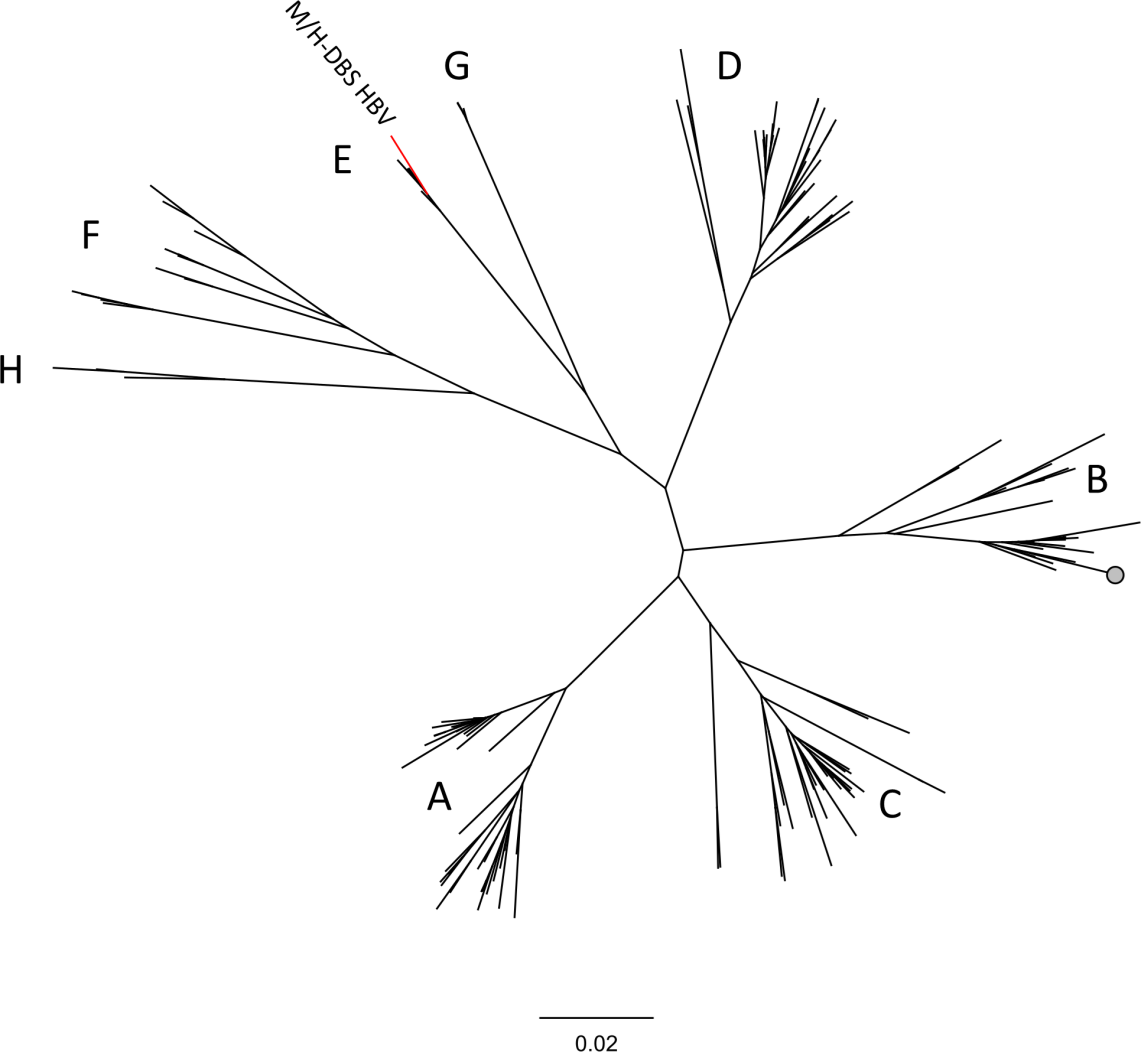
HBV sequences from H and M-DBS cluster phylogenetically with HBV strains from West Africa. The longest contig assembled to HBV was a 542 n.t. segment. This was used as input to create a phylogenetic tree using a neighbor-joining method. Letters correspond to HBV genotype. See S2 Fig for accession numbers corresponding to each HBV used in the analysis.

A single contig from our H-DBS DNA sequencing dataset produced an alignment to an African strain of HBV. This genome was then used to make an indexed reference, and reads from both H-DBS and M-DBS were aligned (Fig 3B). A total of 6 and 2 reads aligned from the H-DBS and M-DBS DNA sequencing dataset, respectively. Reads from both datasets aligned with up to 99% percent pairwise nucleotide identify and combined to cover ~25% of the genome (Table 2). The overlapping reads/contigs from M-DBS and H-DBS shared 100% pairwise nt identity. The mean read length was 142 nucleotides. HBV segregates into 8 distinct genotypes that generally can be distinguished by geographical location [42]. Viruses making up the group Genotype E circulate in West Africa. A phylogenetic analysis using the 542 n.t. sequence from over 150 HBV sequences correctly placed each virus in their appropriate phenotypic group as laid out by Kramvis et al. [42]. This analysis placed the HBV contig identified in this study in Group E with other HBV sequences from West Africa (Fig 5, S2 Fig).

Our initial taxonomic assessment identified multiple contigs aligning to *P*. *falciparum* and various species of parasitic nematodes in both RNA and DNA datasets. Following removal of mosquito and human sequences, >400 reads aligned to *P*. *falciparum* and over 5,000 individual reads produced an alignment to worm genomes. The following criteria was used to remove spurious alignments: 1) Parasite did not produce top hit in Blastn search, 2) low complexity reads (e.g. ATAT repeats), or 3) reads aligned to conserved ribosomal RNA sequences. Following these criteria, 426 reads produced a quality alignment to multiple genes across all chromosomes of the *P*. *falciparum* genome (S2 File), and no nematode parasite reads/contigs were deemed legitimate. The vast majority of reads/contigs that were aligned to the worm index file were removed following a Blastn search, as the top hit produced was to bacterial sequences (S3 File).

### Prevalence

Virus-specific RT-PCR and qPCR was employed to determine the prevalence of GBV-C and HBV from individual H-DBS and M-DBS that made up the NGS pools. We determined that a single H-DBS from one household assessed in Village A was GBV-C positive, resulting in a prevalence of 14.3%. Out of a total of 34 M-DBS, three were deemed positive, resulting in a prevalence of 8.8% in M-DBS collected from the same household (Table 3). A total of 7 H-DBS from 4 separate houses were determined positive for HBV and a total of 17 M-DBS from 8 separate houses also tested positive for HBV, resulting in a prevalence of 7.1% and 13.3%, respectively (Table 3). At least one positive M-DBS was collected from each house that contained at least one positive H-DBS, while not all M-DBS determined positive for HBV were collected in houses with positive H-DBS.

**Table 3:** Prevalence of human viruses by PCR.

	GBV-C		HBV	
	H-DBS	M-DBS	H-DBS	M-DBS
N	7	34	98	127
# Positive	1	3	7	17
Prevalence	14.3%	8.8%	7.1%	13.3%

## Discussion

While efforts to predict pathogen emergence in human populations have improved and become more robust, pathogen emergence remains unpredictable [12]. This highlights the need for vigilant infectious disease surveillance using cost effective, efficient methods for sample acquisition. Hematophagous arthropods have been used to survey wildlife populations for pathogen circulation [43, 44], and human pathogens have been detected in blood meals of these arthropods [29, 45–47]. Using hematophagous arthropods as a sampling method in lieu of direct sampling techniques can be advantageous, but a comparison between the two has never been made. Accordingly, in this study, we sought to improve on existing xenosurveillance methodology while comparing this method to more traditional human blood collection approach, finger-stick blood.

Over 40 households from two villages in northern Liberia were enrolled, resulting in a total of 185 participants (Table 1). From these households, we aspirated a total of 253 mosquitoes, the majority being blood fed *An*. *gambiae*. *An*. *gambiae* mosquitoes are highly anthropophilic [48], and can often be found resting inside houses following the acquisition of a bloodmeal [29, 49], therefore are ideal for xenosurveillance. Moreover, *An*. *gambiae* mosquitoes transmit comparably few pathogens, specifically *Plasmodium sp*., *Wuchereria bancrofti*, and O’nyong nyong virus. This limited number increases the likelihood that pathogen nucleic acids detected by xenosurveillance are derived from the recently fed upon host.

Capturing blood fed mosquitoes from inside houses is less burdensome to the occupants as compared to other direct techniques (e.g. finger-stick blood collection), and presents less of a risk for health workers and researchers. While specialized equipment is necessary (e.g. Insectazookas) and training is required for xenosurveillance, needles, sharps containers, antiseptics, and bandages are not needed, the occupants can continue with their regular activities, and residents do not need to be present when sampling occurs. The non-invasive nature of this technique also facilitates more frequent sampling, resulting in more blood samples collected, thus increasing the possibility of sampling pathogens that occur transiently in the blood. Additionally, no specific training is required to collect and process the mosquitoes and IRB approval is not required, as human blood is not being sampled directly. Furthermore, storing blood spots on FTA cards with RNA Later allows for bypassing cold chain, ensures deactivation of potential pathogens in samples [50], and results in nucleic acid that is a high enough quality to sequence.

To determine if samples collected by xenosurveillance produced data similar to samples collected by finger-stick, we subjected both to NGS on Illumina platforms. A large number of reads were produced in both our RNA and DNA NGS datasets, however, we observed a considerable reduction in reads following quality control and host filtering. Greater than 99% of reads were removed in all sequencing libraries. This is likely due to multiple factors. The RNA NGS pools saw a greater than 90% reduction in reads following removal of PCR duplicates. Because a very low quantity of RNA is recovered from individual DBS, a substantial amount of amplification is required to bring libraries to a usable quantity of nucleic acid. For RNA NGS, increasing the number of DBS per pool would reduce duplicate reads. For DNA NGS, less amplification was required as more DNA is eluted off of individual DBS. This indicated that DNA from mosquito blood-meals was more stable on FTA cards compared to RNA, which allowed us to pool more individual DBS per pool. Predictably, more unique reads were produced from DNA sequencing libraries than RNA sequencing libraries. Substantial losses of reads were observed following filtering of host nucleic acid, indicating most of the sequenced nucleic acid was derived from either humans or mosquitoes, as expected (Fig 2). These reads are not informative for pathogen identification, however they indicate mosquito blood meals were likely taken from humans. Although our in-house depletion strategy worked at clearing our M-DBS RNA sequencing samples of mosquito rRNA, the remainder of the samples would benefit from improved host depletion or pathogen nucleic acid enrichment strategies.

Following quality control and host filtering, an adequate number of reads remained to detect genetic signatures of viruses and parasites. Following further analysis, reads aligning to parasitic worms proved to be largely spurious based on additional bioinformatics scrutiny. This is likely the result of bacterial sequences from our samples aligning to misassemblies in the published genomes. The incorporation of contaminating bacterial sequences into whole genome assemblies is not uncommon [51–53]. As well, Liberia is endemic for lymphatic filariasis and *An*. *gambiae* is a primary vector of *W*. *bancrofti* in West Africa, therefore any reads aligning to this parasite in xenosurveillance samples must be from an infected host or and infected vector [54]. Similarly, multiple reads aligning to *P*. *falciparum* were identified in all sequencing pools, which is expected, however we cannot say with confidence from xenosurveillance samples whether the reads are host or vector derived. Reads aligning to *P*. *falciparum* do highlight the fact that while xenosurveillance is intended to identify any human pathogens present in peripheral blood, not just those transmitted by mosquitoes, these samples can also contribute to routine vector surveillance efforts (e.g. identifying the malaria parasite in local *Anopheles* mosquitoes). While we did not quantify the prevalence of *P*. *falciparum* from individual DBS in this study, the sample collection and storage method would allow for such testing. Furthermore, xenosurveillance can be conducted using other hematophagous arthropods, although a clear understanding of the natural history of the chosen organism and their ability to vector pathogens is crucial when conducting downstream analysis.

Reads from two viruses, GBV-C and HBV, were identified using bioinformatics and molecular methods (Fig 3). Interestingly, although GBV-C, an RNA virus closely related to hepatitis C virus, is not known to be pathogenic, infection with this virus is associated with an increased rate of survival in patients co-infected with either HIV or Ebola virus [41, 55]. As this virus infects and replicates in CD4-positive T cells, we would expect to be able to detect GBV-C RNA in blood [56]. GBV-C has an estimated prevalence between 10–28% in West African countries, which is similar to our findings, albeit derived from a small sample size (Table 3) [41]. Both H and M-DBS, collected from the same household, produced a similar number of reads that aligned with high confidence to GBV-C (Fig 3A) from pooled samples. HBV reads were identified in DNA NGS pools [57]. Chronic HBV infection is the leading cause of chronic hepatitis and hepatocellular carcinoma, and is responsible for up for up to 30% of liver cirrhosis cases globally [58, 59]. Prevalence is highly variable across Africa, but is estimated to be between 4–8% in Liberia as determined by HBV surface antigen, which is congruent with our data (Table 3) [60]. Phylogenetic analysis clearly indicates both viruses are of human origin and from West Africa (Figs 4 and 5). Fewer reads from H and M-DBS aligned to HBV compared to GBV, however substantially more DBS made up the sequencing pool (Fig 3B). As well, the DNA NGS was performed on an Illumina MiSeq, producing substantially fewer overall reads than RNA NGS pools sequenced on an Illumina NextSeq (S2 Table). Nevertheless, these results indicate that NGS can be a sensitive enough technique to detect a pathogen “signal” through host “noise” in complex pooled samples of blood collected by either method. GBV-C and HBV nucleic acid was amplified using qRT-PCR and qPCR, respectively, from individual DBS, showing alternative methods can be used to detect the presence of pathogens in lieu of NGS. Due to similarity in read quality and composition collected from by each method, our data suggest xenosurveillance is a viable alternative for collecting human blood samples used in pathogen surveillance.

Xenosurveillance can be a useful strategy to supplement existing public health efforts in developing regions of the world. As mosquitoes constantly take blood meals from humans, especially in the tropics, the availability of samples is enormous. Xenosurveillance is less invasive and logistically challenging compared to traditional sampling methods. Sample storage is simple and cost effective. As well, sample quality does not appear to suffer from being collected by a mosquito, as there are no substantial differences between samples collected via xenosurveillance or finger-stick. However it remains to be determined if samples collected by xenosurveillance are useful for other means of disease surveillance (e.g. serology). Xenosurveillance is capable of detecting a wide array of pathogens [30], although this study did not reliably detect parasites and bacteria infecting humans. However, zoonotic, viral pathogens are disproportionately likely to reach pandemic emergence, as compared to parasites or bacteria [14]. Recent pandemics of zoonotic viruses, including Ebola virus, Middle East respiratory syndrome coronavirus, and Zika virus support this notion [61–63]. In theory, routine xenosurveillance could provide early warnings by detecting emerging pathogens circulating at low levels in vulnerable populations. As well, xenosurveillance can be targeted to support epidemiological services by collecting engorged mosquitoes in areas where suspected pathogen transmission is occurring [64]. Species specific PCR can be used to identify known or suspected pathogens from xenosurveillance samples, although the use of NGS provides an unbiased detection approach. As the cost of high throughput NGS continues to decrease, large scale sequencing projects are becoming more universal [65]. Furthermore, as field applicable sequencing tools are being further developed and optimized, the use of real-time NGS for surveillance and diagnosis of pathogens is being realized [66–69]. The combination of xenosurveillance and novel sequencing strategies has the potential to bring active, real-time, disease surveillance to resource-poor areas of the world.

## Supporting information

S1 Checklist STROBE checklist. (DOC)

S1 Fig GBV-C phylogenetic tree containing accession values. (TIF)

S2 Fig HBV phylogenetic tree containing accession values. (TIF)

S1 Table Primer sequences used for PCR confirmation of NGS data. (XLSX)

S2 Table NGS read breakdown following quality control and filtering. (XLSX)

S1 File Output of taxonomic assessment program. (XLSX)

S2 File Reads/Contigs aligning to the *P*. *falciparum* genome. (XLSX)

S3 File Description of reads misaligning to parasite genomes. (XLSX)
