# Supplementary figures and images for: Xenosurveillance reflects traditional sampling techniques for the identification of human pathogens: A comparative study in West Africa
Source: PLoS Negl Trop Dis. 2018 Mar 21;12(3):e0006348. doi: 10.1371/journal.pntd.0006348 (PMC5880402; doi:10.1371/journal.pntd.0006348)

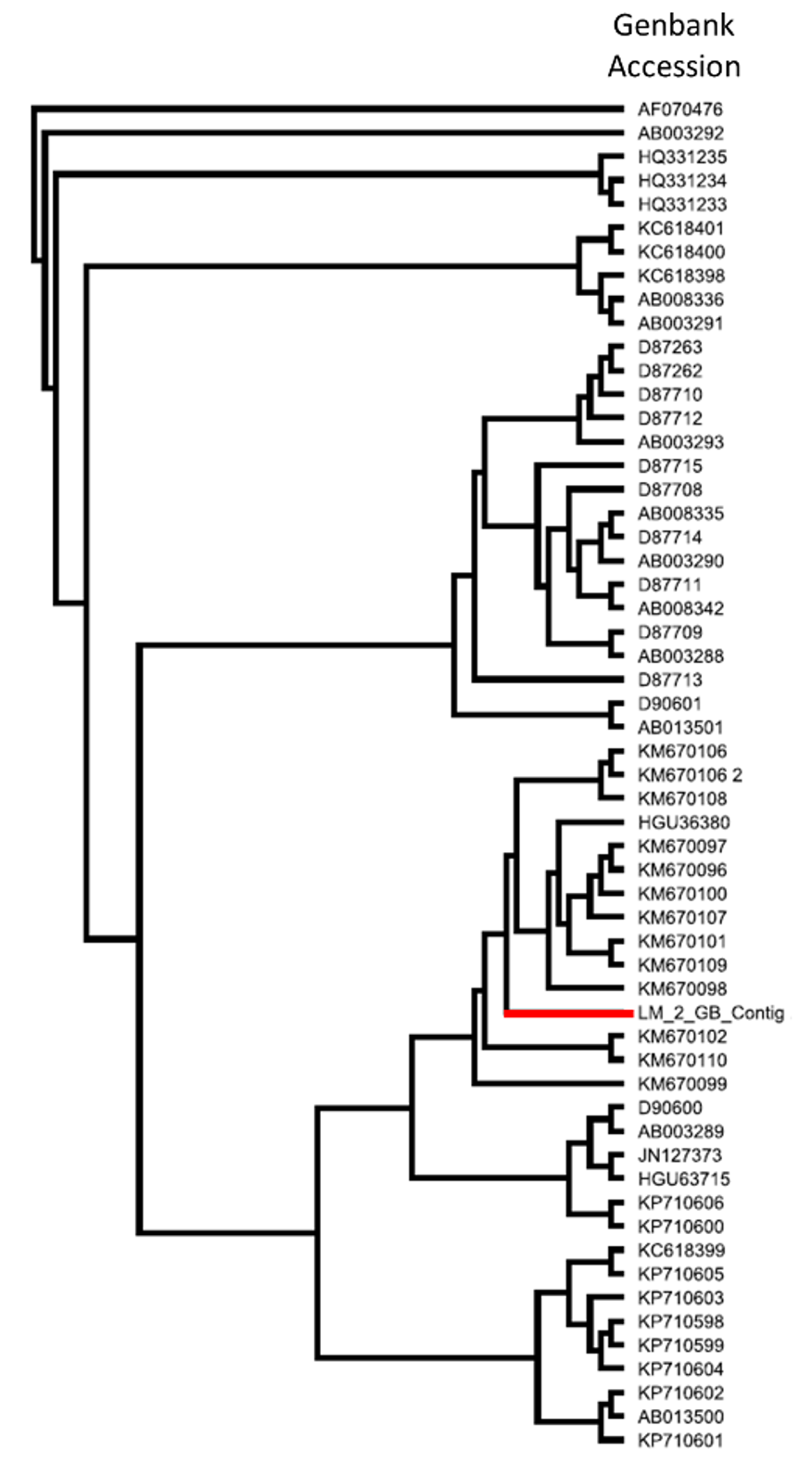

Supplement: S1 Fig — (TIF) [file pntd.0006348.s002.tif]

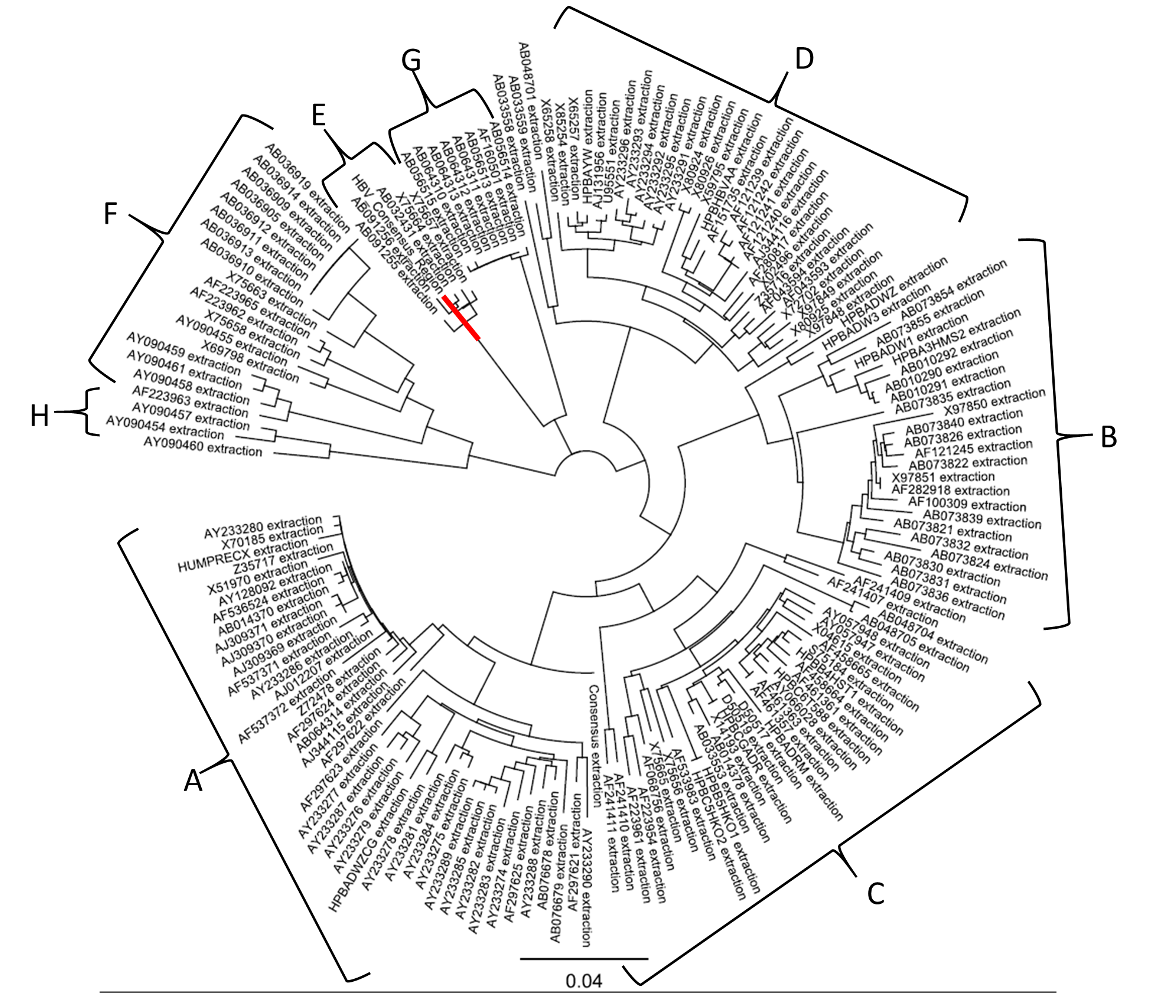

Supplement: S2 Fig — (TIF) [file pntd.0006348.s003.tif]
